# Methodological reflections on health system-oriented assessment of maternity care in 16 hospitals in sub-Saharan Africa: an embedded case study

**DOI:** 10.1093/heapol/czac078

**Published:** 2022-09-10

**Authors:** Anteneh Asefa, Jean-Paul Dossou, Claudia Hanson, Christelle Boyi Hounsou, Gertrude Namazzi, Samuel Meja, Dickson Ally Mkoka, Gottfried Agballa, Josephine Babirye, Aline Semaan, Kristi Sidney Annerstedt, Thérèse Delvaux, Bruno Marchal, Sara Van Belle, Virginia Castellano Pleguezuelo, Lenka Beňová

**Affiliations:** Department of Public Health, Institute of Tropical Medicine, Antwerp, Belgium; Department of Health Policy and Systems, Centre de Recherche en Reproduction Humaine et en Démographie (CERRHUD), Cotonou, Benin; Department of Global Public Health, Karolinska Institutet, Solna, Sweden; Department of Disease Control, London School of Hygiene and Tropical Medicine, London, UK; Department of Health Policy and Systems, Centre de Recherche en Reproduction Humaine et en Démographie (CERRHUD), Cotonou, Benin; Centre of Excellence for Maternal Newborn and Child Health, Department of Health Policy Planning and Management, School of Public Health, Makerere University, Kampala, Uganda; The Centre for Reproductive Health, Kamuzu University of Health Sciences, Blantyre, Malawi; Department of Clinical Nursing, Muhimbili University of Health and Allied Sciences, Dar es Salaam, Tanzania; Department of Health Policy and Systems, Centre de Recherche en Reproduction Humaine et en Démographie (CERRHUD), Cotonou, Benin; Centre of Excellence for Maternal Newborn and Child Health, Department of Health Policy Planning and Management, School of Public Health, Makerere University, Kampala, Uganda; Department of Public Health, Institute of Tropical Medicine, Antwerp, Belgium; Department of Global Public Health, Karolinska Institutet, Solna, Sweden; Department of Public Health, Institute of Tropical Medicine, Antwerp, Belgium; Department of Public Health, Institute of Tropical Medicine, Antwerp, Belgium; Department of Public Health, Institute of Tropical Medicine, Antwerp, Belgium; Department of Public Health, Institute of Tropical Medicine, Antwerp, Belgium; Department of Public Health, Environments and Society, London School of Hygiene and Tropical Medicine, London, UK; Department of Public Health, Institute of Tropical Medicine, Antwerp, Belgium

**Keywords:** Health facility assessment, maternal and newborn health, Benin, Malawi, Tanzania, Uganda

## Abstract

Health facility assessments (HFAs) assessing facilities’ readiness to provide services are well-established. However, HFA questionnaires are typically quantitative and lack depth to understand systems in which health facilities operate—crucial to designing context-oriented interventions. We report lessons from a multiple embedded case study exploring the experiences of HFA data collectors in implementing a novel HFA tool developed using systems thinking approach. We assessed 16 hospitals in four countries (Benin, Malawi, Tanzania and Uganda) as part of a quality improvement implementation research. Our tool was organized in 17 sections and included dimensions of hospital governance, leadership and financing; maternity care standards and procedures; ongoing quality improvement practices; interactions with communities and mapping of the areas related to maternal care. Data for this study were collected using in-depth interviews with senior experts who conducted the HFA in the countries 1–3 months after completion of the HFAs. Data were analysed using the inductive thematic analysis approach. Our HFA faced challenges in logistics (accessing key hospital-based respondents, high turnover of managerial staff and difficulty accessing information considered sensitive in the context) and methodology (response bias, lack of data quality and data entry into an electronic platform). Data elements of governance, leadership and financing were the most affected. Opportunities and strategies adopted aimed at enhancing data collection (building on prior partnerships and understanding local and institutional bureaucracies) and enhancing data richness (identifying respondents with institutional memory, learning from experience and conducting observations at various times). Moreover, HFA data collectors conducted abstraction of records and interviews in a flexible and adaptive way to enhance data quality. Lessons and new skills learned from our HFA could be used as inputs to respond to the growing need of integrating the systems thinking approach in HFA to improve the contextual understanding of operations and structure.

Key messages

- Commonly used health facility assessment (HFA) tools such as the Service Availability and Readiness Assessment and Service Provision Assessment are structured and only quantitative and lack depth to understand the interface between health systems and hospital governance.
- This study reports lessons from implementing a maternity care-focused, mixed methods and flexible HFA conducted in 16 hospitals in four sub-Saharan African countries to inform a quality improvement project.
- Our HFA tool yielded rich data, which were used to design context-oriented maternal and newborn health quality improvement interventions in diverse settings.
- Methodological lessons reported in this study are highly relevant to inform future HFA tools aimed at the measurement of maternal and newborn health service provision and quality of care gaps.

## Introduction

Ensuring high quality of maternal and newborn care is a key health system performance parameter, which involves periodic tracking and evidence-informed actions to achieve universal health coverage targets of the Sustainable Development Goals (SDGs) (Koblinsky *et al.*, 2016; Kruk *et al.*, 2016). Health facility assessment (HFA)—a health system performance assessment tool—is used in diverse settings to periodically assess service availability and facility readiness through the collection of mostly quantitative data not routinely collected through the health management information system (HMIS) (World Health Organization, 2021). In settings with weak HMIS, HFAs are conducted more frequently as health planning and key decisions including financing for health-care delivery largely rely on them (World Health Organization, 2014). In these settings, assessing progress in key milestones, e.g. quality improvement, and ensuring accountability remain challenging (Kruk *et al.*, 2018).

Utilization of maternal and newborn health (MNH) care, particularly during pregnancy and childbirth has risen substantially in sub-Saharan Africa (SSA) over the past decades (Moller *et al.*, 2017; Doctor *et al.*, 2019; Bobo *et al.*, 2021; Tessema *et al.*, 2021). It is now estimated that poor quality of care accounts for more avoidable deaths than non-utilisation of care (Kruk *et al.*, 2018). Therefore, ensuring sound planning and accountability for high-quality MNH care is urgently required at national, sub-national and facility levels to achieve the envisioned SDG targets (Kruk *et al.*, 2018; McArthur *et al.*, 2018; World Health Organization, United Nations Population Fund, 2021).

Understanding the functionality and readiness of health facilities using HFAs is essential in designing effective and context-appropriate MNH quality improvement initiatives (World Health Organization, 2014; 2021; Kruk *et al.*, 2018). Within the MNH health domain, there are various tools for conducting HFAs, including the Service Provision Assessment (SPA) (The DHS Program, 2017), Service Availability and Readiness Assessment (SARA) (World Health Organization, 2015) and the Averting Maternal Death and Disability (AMDD) Emergency Obstetric Care (EmONC) needs assessment (Averting Maternal Death and Disability, 2019). These tools gather data on inputs (infrastructure, equipment and supplies and staffing) and processes in health facilities which provide MNH services and play a paramount role in understanding the overall health system capabilities across various levels and geographic regions (Papanicolas and Smith, 2013). However, some issues remain, such as those related to their ability to comprehensively and rigorously assess key quality parameters of MNH (Brizuela *et al.*, 2019), reliability and external validity and their use as a learning tool to improve the health system within which MNH service provision operates (Sheffel *et al.*, 2018). There is also a paucity of evidence on the extent to which the tools enable a comprehensive assessment of the planning, organization and implementation of MNH services—including financing, governance and leadership mechanisms—in health facilities. Furthermore, despite the frequency with which HFAs are conducted, reflection on HFA methods and lessons learned is sparse.

Our study aims to address some of these gaps through the application of systems thinking in the design and implementation of an in-depth HFA. We posited maternity units as a complex subsystem operating in a complex hospital system (Asefa *et al.*, 2020) and assessed health system components that may have direct or indirect relationships with the provision of MNH services. The objective of our study is to describe and examine the processes of data collection within and across 16 hospitals in four SSA countries and to collate important lessons related to the contextualization of the HFA implemented. To achieve this, we describe the experiences of data collectors who conducted the HFA, identify and discuss methodological lessons and reflect on issues relevant to the analysis and interpretation of other HFAs (SPA, SARA and AMDD EmONC needs assessment tool) (World Health Organization, 2015; The DHS Program, 2017; Averting Maternal Death and Disability, 2019). The outputs of this study could be used to improve the comprehensiveness, rigour and potential of HFAs to inform and strengthen MNH quality improvement initiatives.

## Methods

### The health facility assessment

The HFA was undertaken as part of the Action Leveraging Evidence to Reduce perinatal morTality and morbidity in sub-Saharan Africa (ALERT) study, a 5-year multi-country (Benin, Malawi, Tanzania and Uganda) implementation research project (Akuze *et al.*, 2021). The project aims to design, implement and evaluate an innovative and multidimensional quality improvement intervention to reduce perinatal mortality and is organized in nine work packages, each led by teams of multidisciplinary researchers. In each country, three public and one faith-based hospital providing maternity care services were selected, making a total of 16 hospitals. Detailed description of the hospitals is presented in Supplementary file 1. The HFA was conducted in all 16 hospitals by local senior experts in the field of MNH (HFA data collectors) between December 2020 and April 2021. All HFA data collectors had a master’s degree or higher level of education with backgrounds in clinical training (nursing or doctor of medicine). The HFA data collectors previously worked in their country’s health system for between 7 and 23 years. All HFA data collectors had substantial previous research experience and worked or collaborated with some of the hospitals.

The HFA questionnaire was designed by ALERT researchers after reviewing existing MNH-relevant HFA tools including SPA (The DHS Program, 2017), SARA (World Health Organization, 2015) and EmONC needs assessment (Averting Maternal Death and Disability, 2019). The questionnaire had 17 sections and spanned dimensions relevant to hospital administration (governance, leadership and financing), functioning of hospital pharmacy and laboratory, extraction of aggregate monthly service statistics for 20 maternal and perinatal health indicators, understanding of maternity guidelines and quality improvement processes, observations of infrastructure and service delivery, and a visual representation (map) of the hospital structure in relation to the labour/delivery ward and other spaces relevant to the provision of maternal and newborn care (Dossou *et al.*, 2022).

Of note, Section 3 of the ALERT HFA uniquely assessed governance, leadership and financing functions of hospitals and the dynamics of local health systems with direct and indirect impacts on the hospitals. To assess governance and leadership practices in participating hospitals, the tool included questions on decision-making power, frameworks and processes, both at the hospital and maternity unit levels (micro-level governance actors); hospital stewardship systems at the level of the hospital board, health district and national level (meso- and macro-level governance actors) and engagement of staff, community and other stakeholders (Durán and Saltman, 2015), all capable of affecting the quality of MNH services both directly and indirectly (Hilber *et al.*, 2016). Similarly, questions on sources of financing, user fee administration (if any), hospital and maternity budgeting, setting of user fees for maternal health care (if any), staff incentives and motivation, and financial management and transparency were included to assess financing practices in participating hospitals. Additionally, 18 questions on changes due to the COVID-19 pandemic were added to the tool to gather data on adjustments made to service provision. The ALERT HFA took on average 2–3 days per hospital to administer and required an average of three and up to six respondents per hospital to capture information on the broad range of questions. The HFA questionnaire included multiple-choice questions, open-ended questions and observation notes; responses were collected using REDCap tablet-based application (Harris *et al.*, 2009).

### Study design

We adopted a multiple embedded case study design (Yin, 2003), which allowed us to combine several information sources. We defined the case as the implementation of the ALERT HFA tool in the 16 study sites (hospitals).

### Data collection

The main data collection method was in-depth interviews with the HFA data collectors to explore their experience of using the tool. We held interviews with all four ALERT HFA country teams (three females and three males; one in each Malawi and Tanzania, two in each Benin and Uganda).

The in-depth interviews were conducted 1–3 months after the completion of all HFA data collection virtually over Zoom in English by two senior researchers (L.B. and J.-P.D.) using a semi-structured question guide (Supplementary file 2). Questions focused on areas of planning and preparation to conduct the HFAs, experience with collecting data, data capturing and recording, and HFA data collectors’ perceptions of the HFA tool. The interviews took 1–2 h each, in one or two sessions, and were followed up by email exchange for additional details as necessary. Interviews were recorded, transcribed verbatim and cross-checked by L.B. for accuracy.

### Data analysis

Inductive thematic analysis (Braun and Clarke, 2006) was conducted using NVivo software (QSR International, Version 1.4.1). Initially, reading and rereading of the transcripts was performed by A.A. to deeply understand the data and build a set of initial codes. Additionally, line-by-line coding was done to develop a deeper description of key findings using subcodes. These codes were reviewed by L.B. and were sorted and collated by A.A. to form preliminary themes. The themes were then shared with the co-authors (ALERT project members) for review based on their experience in managing and analysing the HFA data. Finally, the agreed-upon themes along with the findings were shared with participants of the in-depth interviews (HFA data collectors) for respondent validation—member checking (Fereday and Muir-Cochrane, 2006). All quotes used to illustrate our findings are from interviews or email exchanges with HFA data collectors.

### Ethics

The ALERT project received ethical approval from ethics committees of all participating countries and institutes (Akuze *et al.*, 2021). The management team of all study hospitals agreed to be part of the ALERT intervention and received a briefing about the project. Additionally, consent was sought from participants of the in-depth interviews.

## Results

**Figure 1.**
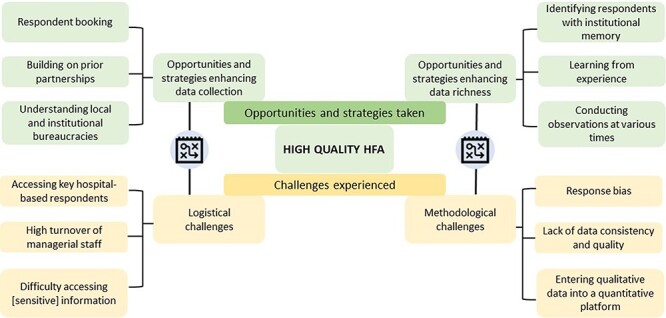
Themes indicating challenges experienced and opportunities and strategies taken during the HFA

Our case study identified a range of reflections and lessons learned in conducting the HFA in hospitals. We present these in four overarching themes (logistical challenges; methodological challenges; opportunities and strategies for enhancing data collection; and opportunities and strategies enhancing data richness and validity) and 12 sub-themes (Figure 1). For convenience and clarity, we present these under two major headings—challenges experienced, and strategies and opportunities taken.

### Challenges experienced

#### Logistical challenges

There were three sub-themes related to challenges HFA data collectors experienced while conducting the HFA from the angle of relational and systemic issues in the hospitals.

##### Accessing key hospital-based respondents

HFA data collectors across the four countries reported that they had difficulty meeting senior hospital staff, such as hospital managers, medical directors and senior administrators. There were instances of postponed appointments with the medical director of one of the hospitals despite sending reminders. Such difficulties negatively impacted the timely completion of the HFA tool, especially the section on hospital governance, leadership and financing. HFA data collectors experienced this in at least one hospital in each country except for Tanzania. Moreover, as those senior staff were the gatekeepers to get permission from and begin the HFA in each hospital, this resulted in delays to starting data collection.

*For one person, for example, I went three times. They told me to come at 9 am. I went before 9 am, they were not there. I stood there until 9 am. I called them after 9am, they did not pick my call. They called me and said ‘oh we postpone the appointment’, gave me another appointment and the same thing happened again. Finally, I did not get the information from them*. (Benin)

HFA data collectors in Malawi and Uganda found it challenging to find focused time with senior staff to complete the HFA. Directors of some hospitals were busy with clinical duties, committee meetings, phone calls and other responsibilities.

*So, myself and a colleague wanted to meet the hospital director. Then when we arrived, we were told that they were going with the team to do hospital rounds. We waited; they did not come back.* (Uganda)

Similarly, some of the hospital administrators were busy with both onsite and offsite meetings, phone calls and activities that required their urgent involvement. Additionally, there were disruptions during interviews, which distracted the attention of key informants and eventually resulted in the postponement of interviews, wasted time and switching to other respondents.

*It was hard for us to meet the administrator. Every time we could knock on their door, they could tell you: they are still busy, give me an hour, give me two hours. The first day we went, we actually left very late waiting for them. Then, the following day was also like that.* (Uganda)

##### High turnover of managerial staff

In several hospitals, hospital directors (position titles varied across the four countries, including medical director, medical officer in charge or medical superintendent) were relatively new in their post. The HFA data collectors were faced with a dilemma of going ahead with interviewing the medical directors alone or devising other strategies to get adequate information for completing the HFA tool, especially the leadership, governance, financing, and infrastructure and supplies sections. However, high turnover of senior administrative and clinical staff made it difficult to locate additional informants.

*…they were fairly junior because the first time we went in December, the one who was in charge had been in the position for less than a year. When we went in January, they had gone for specialization. So, the one who replaced them was basically a month into the position. They have been working at the hospital as a medical officer for six months or so, but in the administrative position, only for a month. The other medical officers have not been there for a long time.* (Malawi)

##### Difficulty accessing information considered sensitive

HFA data collectors reported challenges with access to information considered sensitive despite presenting official permission letters, ethical clearance, synopsis of the ALERT project and letter from the hospital-in-charge. This problem was particularly pronounced for administrative and financial data in Benin and Tanzania. In one of the hospitals, the head of administration remained reluctant to provide financial information, even after being given direct orders from the hospital director. Additionally, the fact that all participating hospitals used different approaches of financial planning and management made the process of completing the financing section of the HFA more challenging.

*So, the administrative head came in the other office when I was discussing with the director and the director asked them [administrative head] to bring [financial] information and they were surprised by the request of the director, and the director repeated again: ‘yes, I told you to bring the information’. At this moment that the [administrative director] went to their office and brought the information about the financial balance.* (Benin)

Moreover, HFA data collectors experienced that some hospitals request women and their families to buy supplies despite the existence of free maternal health-care policies and health workers were reluctant to talk about that.

*It is known that from the policy, you know, in this government facility services for the mothers and newborns are free. So, sometimes if you go and ask the hospital informant that ‘can you tell me, are mothers paying?’ It is like, hmm, why are you asking, you know that…you see?* (Tanzania)

Additionally, there was reluctance to provide data on numbers of maternal deaths in hospitals in Uganda. It was reported that some of the staff wanted to have a direct verbal instruction from the hospital director before sharing these data.

*There was maternal and perinatal death surveillance and response auditing the death. So, they [department heads] refused to show us their maternal death review forms. They said unless we want the plain [blank] ones, which are public documents that you can get on the web, they cannot give us their review forms; you know because I think in Uganda, maternal death is so sensitive that at one time, some health workers have been arrested over that.* (Uganda)

Participants also reported that staff who provided information following the instruction of their supervisors regarded HFA data collectors as government supervisors because disclosure of sensitive information to outsiders was rare in their experience.

#### Methodological challenges

During data collection, HFA data collectors described being faced with methodological challenges, which could affect the accuracy, completeness and contextual richness of the collected data. These challenges stemmed from the nature of the HFA tool itself and the types and composition of key informants in the participating hospitals. We present the methodological issues under three sub-themes.

##### Social desirability bias

HFA data collectors reported issues of response bias following some of the questions—especially those targeting governance, quality improvement and human resources—collected from senior hospital administrators. Some of the HFA data collectors had prior collegial or student–teacher relationships with hospital-based respondents or long-term relationships with the hospital itself (e.g. involvement with national committees or previous research projects). According to the HFA data collectors, staff who were their [HFA data collectors’] former students and research graduates might have responded in a way to please them or show their competence. HFA data collectors also encountered former colleagues as their key informants during the HFA and felt that such pre-existing acquaintance might have introduced social desirability bias.

*…I felt the information I was getting was somehow not real, I had a feeling this is something they [former students] want me to hear. You get what I mean, sort of, as if I went there and it is a direct assessment of how they are doing now that they have graduated. I had that feeling.* (Malawi)

##### Lack of quantitative data consistency and quality

HFA data collectors from all four countries reported that some of the routine service statistics included in the HFA questionnaire were not available in the hospitals’ recording and reporting systems, either completely not collected or missing for certain months. The post hoc communication with HFA data collectors helped the ALERT data managers and analysts distinguish between true zero values, indicators missing from the hospital records completely, indicators representing procedures which were not practiced at all, and data missing due to data entry error (which was later corrected or completed as needed). For example, in Uganda, the indicator on the number of assisted vaginal births was entered as ‘zero’, and the reason for this was that the practice of ‘vacuum extraction was no longer recommended/allowed in Uganda, and forceps delivery was stopped long time ago’.

In some hospitals, the district health information software (DHIS2) was helpful in collating relevant indicators in one place, making it easier for HFA data collectors to retrieve data, specifically in the case of simple indicators such as the total number of antenatal visits, deliveries and stillbirths. However, the completeness and accuracy of routinely collected DHIS2 data for certain indicators, such as preterm births and episiotomies, were low and not useful for the HFA data collection.

*…about the statistics, it was difficult for them to give us because they do not have…it is not routinely collected. There is some information that they do not collect daily.* (Benin)

HFA data collectors then attempted to collate relevant data from routine registers and record books in the maternity ward, with several challenges. For example, some registers were missing completely in one hospital in Uganda. HFA data collectors reported being surprised that there was no system capturing complete documentation of deaths which happened in some of the hospitals’ maternity wards.

*It was difficult to get statistical information because they were quite scattered because they do the kind of paperwork in a way. They fill information in different books and then you have to go through these books.* (Tanzania)

##### Entering qualitative data into a quantitative platform

During the data collection, HFA data collectors entered quantitative data (single-response dropdown, yes/no and multiple-choice questions) and short responses from open-ended questions directly into the REDCap platform on tablets. Long responses to open-ended questions were recorded separately (in writing on a piece of paper or voice recording with permission) and later transcribed into the REDCap platform. Questions targeting topics of hospital governance, leadership and financing dimensions were the most difficult sections for combining data input (quantitative and qualitative) while interviewing.

*…it was difficult doing the interview and filling REDCap. So, what we did, we had a hard copy of the tool, but also, we requested them [staff] permission to record the interview so that we make sure that every information is captured and we do not miss anything.* (Tanzania)

Navigating the REDCap for quantitative data and short responses to open-ended questions and switching from REDCap to audio-recording for long responses were even more challenging when respondents interrupted interviews for urgent matters. HFA data collectors also mentioned that there were times when they had technical troubles uploading long text responses into REDCap (some of these responses were longer than 1000 words). Additionally, long text entries were sometimes rendered illegible in REDCap, meaning that responses had to be entered twice or even thrice, potentially compromising the richness of final responses recorded.

### Opportunities and strategies taken

#### Opportunities and strategies enhancing data collection

Various techniques for facilitating the HFA were taken by data collectors across the four countries, ranging from accessing key staff, approaching department/unit heads, extracting quantitative data and conducting independent observations. We present these under three sub-themes.

##### Respondent booking

HFA data collectors reported various approaches to liaising with medical directors and hospital administrators. While HFA data collectors went in person to hand over a letter of cooperation to the hospital director of some of the hospitals, they booked an appointment in advance (via email or phone call) for the majority of the hospitals. In scenarios where HFA data collectors were able to find contact information of department/unit heads, they also communicated with them in advance so that they had a smoother start to the data collection upon approval from hospital in-charges.

*First, we had phone conversations with each of the contacts at each hospital; mostly, we contacted the district medical officers who are in charge of these hospitals and this phone conversation was backed up by emails back and forth or sometimes WhatsApp messages to arrange for a perfect date to have the facility assessment.* (Malawi)

Most of the hospitals assessed had limited human resources, especially in the laboratory, pharmacy and quality assurance departments. This meant that accessing key staff required a strategy, especially if the data collection period coincided with staff annual leave or absence due to training. In response, advance agreement with respondents helped HFA data collectors schedule visits both across the various departments in the same hospital and across the various hospitals assessed.

*So, you need to plan in advance at least to inform and prepare them; we have to meet maybe the laboratory in charge or the in charge of the pharmacy… ., you might go there and you will find that the one who is in the office is not an experienced person, maybe an acting [interim].* (Tanzania)

In hospitals where it was possible to communicate with key staff in advance, the level of readiness among staff to host HFA data collectors and engage in the HFA was better. In some cases, hospital directors along with department/unit heads and key staff waited for HFA data collectors for a briefing meeting during which the HFA data collectors explained the purpose and processes of the HFA. There were occasions when HFA data collectors scheduled after-hours or weekend appointments with key hospital respondents to complete the HFA.

##### Building on prior partnerships

HFA data collectors reported that previous collaborations (research, training and implementation) with some of the hospitals were very helpful to gain support during the HFA.

*Previously we [HFA data collectors] had a pre-term initiative study in six hospitals; out of the six, four are the ones under the ALERT project.* (Uganda)

##### Understanding local and institutional bureaucracies

This study identified that the process of seeking approval from hospital directors and permission from department/unit heads was different across hospitals and countries. In order to gain access to informants, it was critical for the HFA data collectors to understand the organizational culture of each hospital.

*…for Hospital X, the director sent the letter to the head of the maternity unit who writes a memo, a service memo, that we use as an authorization to discuss with the people in the unit.* (Benin)

From the angle of power dynamics, especially within the district health systems, HFA data collectors reported that a courtesy visit or call to district medical officers was instrumental to gain the buy-in of key hospital managers. HFA data collectors also reported that they had opportunities to meet with some of the hospital directors at the district health departments and that this made the official process even faster.

#### Opportunities and strategies enhancing data richness and validity

In this theme, we describe the strategies HFA data collectors devised to collect richer and valid data in response to the various logistical and methodological challenges they encountered.

##### Identifying respondents with institutional memory

In the absence of key hospital staff and in circumstances where staff were very new to their positions, HFA data collectors asked for senior staff who know the organizational culture of the hospitals. Similarly, some recently appointed hospital directors also proposed senior staff who worked for several years in their hospital to provide data needed. The approach was advantageous to collect rich data on the history of the hospitals and contemporary governance, leadership and financing issues, especially in hospitals with a rapid turnover of managers and medical officers.

*…we requested for permission of the hospital in charge, we have this kind of question about governance, but we need to clarify. They said, “I’m very new to this position and you can get most of this information from the in charge of the labour ward who was leading this hospital for many years*.*”* (Tanzania)

Additionally, some of the hospitals, especially in Benin, employ communications staff. These staff reportedly stay in their hospitals for longer periods and thus knew important information about how the hospital operates. Accordingly, in circumstances where adequate information could not be gathered from recently appointed hospital directors, the communications staff were interviewed.

##### Conducting observations at various times

This sub-theme focuses on sections of the ALERT HFA involving observations of the workflow and processes in the hospitals. HFA data collectors made visits to some of the hospitals at night and on weekends to interview key respondents who were not available during working hours, especially in maternity units. During these visits, HFA data collectors found an appreciable difference in terms of lower staff availability and support provided to maternity clients. Based on this experience, they noted that data collected during working hours will not accurately reflect how the hospitals and maternity wards operate on a 24/7 basis.

*…We visited two hospitals during weekends and weekdays… … during weekdays, there were four health workers in the labour ward according to the roster. But, when we visited the same hospital at the weekend, only two health workers were there while the mothers were many. Only a few staff were there in the hospital, including medical attendants and those who help the nurses such as cleaners.* (Tanzania)

##### Learning from experience—starting from the nearest hospital

Across the four countries, the sequence in which the hospitals were approached varied. Some teams started from the farthest hospital with the aim of (1) making the process more time- and resource-efficient, (2) pre-informing the other hospitals on their way to the farthest hospital and (3) scheduling for a second visit to the closest hospitals in the event of failing to complete the fieldwork within the anticipated time. Eventually, one of the HFA data collection teams had to also travel to the farthest hospitals twice to ensure data completeness. On the other hand, other teams started from the nearest hospital aiming to learn from the process and make necessary adjustments before they travelled farther. Although both approaches had their merits and demerits, the broader HFA data collection team (from all four countries and international collaborators) finally concluded that starting from the geographically nearest hospital is helpful to learn from the processes involved and challenges experienced and to devise and share strategies for addressing these.

## Discussion

In this paper, we presented a reflection on methodological and logistical challenges experienced, strategies taken and lessons learned from an HFA focused on MNH provision in 16 low-income setting hospitals. Despite the frequency with which HFAs are conducted, reflections on HFA methods and lessons learned are sparse. While this was not originally a part of the study, we derived a high value of conducting such reflective self-research shortly after conducting the HFA exercise within the ALERT project. Our study provides lessons which could be used to improve the value and richness of HFA data to enable a systems-oriented understanding of health facilities’ readiness to provide high-quality MNH services. Cognizant of the complexity and dynamicity of hospitals (Barasa *et al.*, 2017), experiences from success stories and implementation challenges of the ALERT HFA tool will also be key to strengthening research and implementation in the field of MNH. As the ALERT HFA assessed responsive actions taken in MNH care organization due to the COVID-19 pandemic, lessons learned from its administration can contribute to designing rigorous HFAs capable of capturing facilities’ resilience and response to shocks and crises (Kruk *et al.*, 2016).

What makes our case study unique is its post hoc design to explore HFA data collectors’ experiences of assessing governance structures and practices that directly or indirectly influence the provision of MNH services. Lack of institutional memory and of routine documentation and reporting of governance functions in almost all assessed hospitals required us to triangulate information from various sources. Improving the capacity of HFA tools contributes towards evidence-based hospital leadership and governance practices for better MNH performance in resource-constrained contexts (Gilson and Agyepong, 2018). It is also key to strengthening accountability mechanisms to improve quality of care and reduce preventable complications and mortalities in MNH settings (Hilber *et al.*, 2016).

In light of the lack of evidence on assessing governance practices in hospitals (Pyone *et al.*, 2017), contextualizing existing HFA tools such as SARA and SPA to include leadership and governance practices in hospitals and their MNH settings is practical and instrumental. Likewise, linking hospital-based HFAs with district health system data or including district-level key informants in HFAs could help to collect comprehensive data on governance, leadership and financing practices from a health system perspective (Mikkelsen-Lopez *et al.*, 2011). Furthermore, in the light of the lack of standardized financial planning and budgeting in low-resource-setting hospitals (Barasa *et al.*, 2017; Kairu *et al.*, 2021), including flexible and open-ended questions assessing hospital financing practices in HFA tools and learning from the data collection process is imperative.

However, as our HFA tool captured more dimensions than tools such as the SARA and SPA, it is unlikely that the critical steps our HFA data collectors used to achieve high data completeness, richness and accuracy—such as flexible duration and timing of fieldwork and multiple visits to hospitals—can be achieved in large-scale HFAs or those covering multiple services. Additionally, the open-ended nature of some questions in the ALERT HFA tool were not understood and interpreted in the same way by all HFA data collectors and hospital informants. To address such limitations and enhance the added value of the mixed methods nature of HFAs in collecting rich data, pretesting HFA questionnaires plays a crucial role (World Health Organization, 2015; Tuckerman *et al.*, 2020). This includes understanding the culture of communication and local shared ‘jargons’ in advance and making clarifications during data collection (World Health Organization, 2015).

As observed in our case study, collecting data only during regular working hours could lead to an incorrect understanding of the patterns of service delivery because client volumes, staff availability and provider–client relationships vary significantly between regular and non-regular working hours (Manu *et al.*, 2018). In some of the ALERT hospitals, HFA data collectors conducted the HFA during weekends and night-time. Reflecting on this specific experience was a critical step to learn health systems within which the hospitals operate (Sheikh and Abimbola, 2021) and identify key areas for perinatal care quality improvement as part of the ALERT project (Akuze *et al.*, 2021). Accordingly, future HFAs in resource-limited settings should consider such differences to understand the patterns of service provision and actual client volumes of facilities and identify key gaps in the effective coverage of life-saving MNH services (Brizuela *et al.*, 2019; Tomlin *et al.*, 2020) (Allen *et al.*, 2017).

Issues in the quality and completeness of routine service statistics, such as those experienced in our case study, are complex and may only be detected at the data-cleaning stage. These could be partly reduced by conducting data-quality checks throughout the HFA process, including second-time visit to health facilities (World Health Organization, 2014; 2015). Accordingly, post hoc communication between HFA data collectors and health facilities should be an integral component of HFA planning and implementation not only to improve the quality of data collected but also to enhance future data recording and reporting in health facilities (Brizuela *et al.*, 2019; Lee *et al.*, 2021). However, such practices are time-consuming for both health workers and HFA data collectors and must therefore be budgeted for and adapted to the context.

### Strengths and limitations

As our HFA was conducted by senior experts and in-depth interviews with them were conducted shortly after fieldwork, the study captures rich lessons learned from a robust HFA that could be used in future HFAs aimed at MNH quality improvement initiatives. Additionally, having researchers (ALERT project members) as participants (HFA data collectors) in our study also adds credibility and trustworthiness to the study findings. Exploring data collectors’ experiences of administering HFAs is an underutilized but resourceful exercise to enhance a rigorous understanding of system-wide issues affecting the provision of services in health facilities. Our study also benefited from exploring contextual nuances during cleaning and preliminary analysis of the HFA data that allowed deep understanding of the HFA’s gaps and challenges and the usefulness of HFA data collected to the quality improvement project at hand.

Our study was prone to response bias that primarily originates from health workers who provided information during the actual HFA, thereby affecting the experiences of HFA data collectors (participants of this study) and their responses in in-depth interviews. Our HFA attempted to minimize such biases by triangulating quantitative and qualitative data collected from various sources that captured key information in the hospitals assessed. Our study is also limited in terms of making an in-depth exploration of lessons learned during the analysis and interpretation of the HFA data, which will be addressed by forthcoming studies from the ALERT project. Future HFAs aiming at a contextual understanding of health facilities’ readiness for MNH care provision in low-resource settings could enhance methodological rigour building on the lessons learned from and the gaps identified by our study.

## Conclusion

This study presents experiences from applying in-depth, mixed methods HFAs for the purpose of informing MNH quality improvement initiatives in four SSA countries. HFA data collectors experienced that hospitals in general and their MNH services in particular present a case of complex and interlinked dimensions, located in the health system and steeped in the organizational history, which are challenging to capture with a survey tool. The extent to which the ALERT HFA tool succeeded in collecting relevant information about the participating hospitals was greatly aided by the pre-established relationship of trust and the shared purpose between hospitals and the ALERT project to improve intrapartum care quality. Additionally, flexibility in terms of logistical arrangements and seniority of the HFA data collectors enlarged the scope of dimensions for which data were collected. The expanded scope of this HFA exercise is unlikely to be possible in more general health facility surveys, such as the SPA or SARA, nor in routine HMISs capturing service availability and provision. However, methodological lessons learned in conducting a mixed methods HFA are highly relevant to HFAs focusing on the measurement of MNH service provision and quality.

## Supplementary Material

czac078_Supp

## Figures and Tables

**Table:** 

**Role**	**Author initials**
Conception or design of the work	A.A., L.B., J.-P.D., C.H.
Data collection	L.B., J.-P.D.
Data analysis and interpretation	A.A., L.B.
Drafting the article	A.A., L.B.
Critical revision of the article	A.A., L.B., J.-P.D., C.H., C.B.H., G.N., S.M., D.A.M., G.A., J.B., A.S., K.S.A., T.D., B.M., S.V.B., V.C.P.
Final approval of the version to be submitted	A.A., L.B., J.-P.D., C.H., C.B.H., G.N., S.M., D.A.M., G.A., J.B., A.S., K.S.A., T.D., B.M., S.V.B., V.C.P.

## Reflexivity statement

The authors include ten females and six males based in six countries with varying levels of seniority. Seven authors, including one joint first author, are from the four countries where the Action Leveraging Evidence to Reduce perinatal morTality and morbidity in sub-Saharan Africa intervention is implemented (Benin, Malawi, Tanzania and Uganda). Six co-authors from the four countries were also study participants, contributing to the trustworthiness of the study’s findings. The authors’ specialities include health systems research, global and local maternal health, demography and health policy. All authors have extensive experience conducting health systems and maternal health research in sub-Saharan Africa, using quantitative, qualitative and mixed methods approaches.
