# Supplementary material for: Methodological reflections on health system-oriented assessment of maternity care in 16 hospitals in sub-Saharan Africa: an embedded case study
Source: Health Policy Plan. 2022 Sep 10;37(10):1257–66. doi: 10.1093/heapol/czac078 (PMC9661265; doi:10.1093/heapol/czac078)
Supplement: czac078_Supp [file czac078_supp.zip › Supplementary file 1.docx]

**Supplementary file 1. Characteristics of the 16 participating hospitals and maternity wards before the COVID-19 pandemic**

| **Country** | **BENIN** | | | | **MALAWI** | | | |
| --- | --- | --- | --- | --- | --- | --- | --- | --- |
| **Hospital** | **BN1** | **BN2** | **BN3** | **BN4** | **MW1** | **MW2** | **MW3** | **MW4** |
| **Hospital characteristics** | | | | | | | | |
| Operating authority | Public | Public | Public | Private | Public | Private | Public | Public |
| Established between | 2000-2005 | 1985-1990 | 1960-1965 | 1995-2000 | 1985-1990 | 1955-1960 | 2000-2005 | 1985-1990 |
| Total number of beds | 134 | 362 | 648 | 68 | 222 | 290 | 100 | 293 |
| Catchment population (in 000s) | 410 | 2805 | 11 880 | 1720 | 630 | 250 | 550 | 685 |
| **Maternity services provided** | | | | | | | | |
| Routine outpatient ANC | Yes | Yes | Yes | Yes | Yes | Yes | Yes | Yes |
| Childbirth/caesarean sections | Yes | Yes | Yes | Yes | Yes | Yes | Yes | Yes |
| Routine outpatient PNC | Yes | Yes | Yes | Yes | Yes | Yes | Yes | Yes |
| Private maternity care^†^ | No | No | No | No | No | Yes | No | No |
| Dedicated theatre for caesarean section | Yes | Yes | Yes | Yes | No | No | Yes | No |
| HDU or ICU for women | Yes | Yes | Yes | No | Yes | Yes | No | No |
| NICU (year NICU added) | Yes (2019) | Yes (1990) | Yes (1978) | Yes (1996) | No | No | No | No |
| **Maternity ward characteristics** | | | | | | | | |
| Number of outpatient ANC visits (2019) | 2588 | 5446 | 4615 | 7045 | 5885 | 4625 | 12 336 | 7791 |
| Number of deliveries (2019) | 2088 | 5740 | 2605 | 2186 | 7363 | 3276 | 6373 | 7791 |
| Perinatal mortality rate* (2019) | -- | 78 | -- | 48 | 51 | 17 | 34 | 19 |
| Number of beds for second stage of labour | 3 | 10 | 4 | 4 | 4 | 6 | 5 | 9 |
| Frequency of women exceeding labour beds | Once a week | Every day | Once a week | Every day | Once a month | Few times per year | -- | -- |
| Number of medical professionals working per shift on weekday daytime** | 6 | 7 | 18 | 3 | 14 | 10 | 13 | -- |
| LOS after uncomplicated vaginal birth as per usual practice | -- | 1 day | 24 hours | 24 hours | -- | 24 hours | 1 day | 24 hours |
| LOS (minimum) after caesarean section as per usual practice | 7 days | 3 days | 4 days | 3 days | 3 days | 3 days | 3 days | 3 days |
| User fees for maternity care | Yes (with exception of caesarean sections) | Yes (with exception of caesarean sections) | Yes (with exception of caesarean sections) | Yes (with exception of caesarean sections) | No | Yes | No | No |

**Supplementary file 1 (cont.) Characteristics of the 16 participating hospitals and maternity wards before the COVID-19 pandemic**

| **Country** | **TANZANIA** | | | | **UGANDA** | | | |
| --- | --- | --- | --- | --- | --- | --- | --- | --- |
| **Hospital** | **TZ1** | **TZ2** | **TZ3** | **TZ4** | **UG1** | **UG2** | **UG3** | **UG4** |
| **Hospital characteristics** | | | | | | | | |
| Operating authority | Private | Public | Public | Public | Public | Private | Public | Public |
| Established between | 1925-1930 | 1950-1955 | 1950-1955 | 1955-1960 | 1930-1935 | 1910-1915 | 1965-1970 | 1965-1970 |
| Total number of beds | 306 | 207 | 138 | 280 | 500 | 160 | 100 | 100 |
| Catchment population (in 000s) | 160 | 28 | 162 | 95 | 4000 | 20 | 2000 | 400 |
| **Maternity services and infrastructure** | | | | | | | | |
| Routine outpatient ANC | Yes | Yes | Yes | Yes | Yes | Yes | Yes | Yes |
| Childbirth/caesarean sections | Yes | Yes | Yes | Yes | Yes | Yes | Yes | Yes |
| Routine outpatient PNC | Yes | Yes | Yes | Yes | Yes | Yes | Yes | Yes |
| Private maternity care | No | No | No | No | Yes | Yes | No | No |
| Dedicated theatre for caesarean section | No | No | No | No | No | No | No | No |
| HDU or ICU for women | No | No | No | No | No | No | No | No |
| NICU (year NICU added) | Yes (2013) | No | Yes (2012) | Yes (2015) | Yes (2020) | Yes^ (2016) | Yes (2020) | Yes^ (2016) |
| **Maternity ward characteristics** | | | | | | | | |
| Number of outpatient ANC visits (2019) | 4469 | 5108 | 3770 | -- | 10 951 | 5855 | 15 622 | 10 070 |
| Number of deliveries (2019) | 2048 | 4788 | 2242 | 2981 | 6070 | 1265 | 7169 | 3531 |
| Perinatal mortality rate* (2019) | 49 | 40 | 44 | 81 | 62 | 115 | 52 | 54 |
| Number of beds for 2nd stage of labour | 4 | 4 | 6 | 5 | 6 | 6 | 6 | 2 |
| Frequency of women exceeding labour beds | Few times per year | Once a week | Once a week | Few times per year | Few times per year | Few times per year | Every day | Every day |
| Number of medical professionals working per shift on weekday daytime** | 3 | 3 | 3 | 5 | 6 | 6 | 4 | 2 |
| LOS after uncomplicated vaginal birth as per usual practice | 12 hours | 12 hours | 12 hours | 12-24 hours; discharge rounds take place once daily | 12 hours | 24 hours | 12 hours | 12 hours |
| LOS (minimum) after caesarean section as per usual practice | 48 hours | 48 hours | 72 hours | 48 hours | 72 hours | 3 days | 72 hours | 4 days |
| User fees for maternity care | Yes | No, but women asked to bring materials/supplies | No, but women asked to bring materials/supplies | No, but women asked to bring materials/supplies | No (with exception of patients in private maternity ward) | Yes | No | No |

^†^Private maternity care refers to the availability of an optional private services such as a private labour ward or private room for postnatal care. The package could include supplementary services such as closer monitoring and provision of drinking water.

*Perinatal mortality rate is calculated as the number of stillbirths and early neonatal deaths per 1,000 births

**Medical professionals include gynaecologists, midwives, nurses, anaesthetists, interns and doctors in training, clinical officers, medical officers, assistant clinical officers, patient attendant.

^Neonatal special care unit

Abbreviations: Antenatal care (ANC); Length of stay (LOS); Postnatal care (PNC); High-dependency unit (HDU); Intensive care unit (ICU); Neonatal intensive care unit (NICU); Human immunodeficiency virus (HIV)

(--) indicates missing data
