# Supplementary material for: Methodological reflections on health system-oriented assessment of maternity care in 16 hospitals in sub-Saharan Africa: an embedded case study
Source: Health Policy Plan. 2022 Sep 10;37(10):1257–66. doi: 10.1093/heapol/czac078 (PMC9661265; doi:10.1093/heapol/czac078)
Supplement: czac078_Supp [file czac078_supp.zip › Supplementary file 2.docx]

**Question Guide**

**Reflections on WP4 Health Facility Assessments**

Debriefing session supported by:

WP4 local data collector name:

WP4 local data collector gender: M / F

Date of debriefing: ____/ _______/ 2021

Hospital Name:____________________ Country: BE/MW/UG/TZ

Date(s) of hospital visit:

*Information:*

*Thank you for your time today in sharing your experience collecting data for the HFA. The information you provide will be used internally by the Consortium to understand the participating hospitals, and to improve on the HFA tool in the future. It will not be used to evaluate your performance or the one of your team. Will you give me a permission to record this conversation?*

*This conversation has two objectives:*

1. *To better understand the notes and data on the Health Facility Form & get to know the hospitals better, on the basis of what you knew about the hospital and your country context, what you have learned during the field visits and the data collection process, your analytical impressions and reflections at this stage in the research process.*
2. *To understand how the HFA form is useful, what are some of the challenges in completing it.*

**SECTION 1. Preparation for visit and arrival**

1.1 How was the process of getting prepared and planning the visit to this hospital?

Probes:

- In terms of deciding about who will collect the data with you, and what will be your role in the team? Who were the people involved in collecting data for this hospital?
- In terms of mastering the tool and its objectives?
- In terms of deciding the order of visits to hospitals? For instance, among the 4 ALERT hospitals in your country, this hospital was which number to be visited by the ALERT team? Why? Which number for you personally (in terms of order, and out of how many)?
- In terms of mastering the RedCap data entry?
- In terms of getting prepared for the logistics?
- In terms of communicating your visit with the hospital stakeholders? Did you bring any information/materials with you?
- In terms of communicating your visit with the ALERT consortium stakeholders? For instance, did you organise a session with key people on the ALERT project before or during your visit? If yes, tell me more about it.

1.2 What can you share about your arrival to the hospital on the first day?

Probes:

- In terms of previous experiences with this hospital? For instance, have you been to this hospital before? (if yes, how long ago?) For what purpose (internship, clinical work, administrative work, supervision, research?)
- Were you expected/awaited? Who received you? How?
- What was your first impression with the hospital as a whole?

**SECTION 2. Hospital in general**

2.1 What can you tell us about the location of this hospital? What is your reflection about what this particular location means for its performance?

Probes:

- How is the road to access it? How isolated is it from most of the population deserved?

2.2 Can you talk me through the building and what is where (debrief from the maps)?

Probes:

- How would women in labour get from the main entrance to the labour ward? Was this clearly sign-posted?
- What were the COVID measures in place during your visits?

2.3 How are the infrastructures of this hospital in general?

Probes:

- Are they rather new/old? Well or poorly maintained? Is this a (renewing) hospital or a rather abandoned hospital?

**SECTION 3. Maternity ward**

3.1 Who showed you around the maternity ward? Name all people.

3.2 Were these informants collaborative?

3.3 What was your impression and learning of the maternity ward?

Probes:

- What pleasantly surprised you? How?
- What did not seem to be going right? How?
- What was your impressions/reflections/questions on how the maternity ward staff (doctors, nurses, midwives) were interacting with patients? Companions? Visitors?

3.4 Among the maternity wards you have seen in your career in your country, how would you rate your impression of this maternity ward (on a scale of 0-least to 10-most) in terms of:

1. Effective organisation of patient flow? [enter number: _______] Why?
2. Sense of orderly work, staff seemingly knowing what they are doing? [enter number: _______] Why?
3. Cleanliness? [enter number: _______] Why?
4. Patient volumes? [enter number: _______] Why?
5. Anything else?

3.5 Would you (or your sister/wife) be happy to seek care in this hospital?

- For a fever? Why?
- For antenatal care? Why?
- For childbirth? Why?

**SECTION 4. Data collection reflections**

4.1 Can you tell me more about the data collection tool and its different sections?

Probes:

- Was there a part that was most difficult to collect? Why?
- Was there a part that was easiest to collect? Why?
- Which part of the questionnaire did you complete first? With whom? How did it go?
- In which order did you complete the form?
- Was this your choice or that of the hospital staff (or their availability)?
- What is important, what is perhaps less important in this tool? Why?

4.2 Tell me more about the time you had available to collect the data for this assessment? Did you feel you had enough time to conduct the assessment? Why?

4.3 What was the key events during the data collect process in this hospital?

Probes:

- Any turning point in the relationship with the informant?
- Any incident or accident?
- Any interview and event observed that influenced a lot your understanding of this hospital?

4.4 What can you share about the informants in this hospital?

Probes:

- How were you received? How adequate were the informants? Did you feel you get the right people to have the right answers to your question? What were there attitude, general behaviour, emotions, feeling during the interviews and while sharing the information with you? For instance were they collaborative, happy, forthcoming, feeling forced, bothered, afraid, concerned, reluctant..?

During your visit, did you ever feel that:

- The people who should have known the answer to specific questions did not know?
- People were not forthcoming with information/data? If yes, why?

4.5 What can you share about the content of the information you collected?

Probes:

- How do you feel about the completeness of the data collection for this tool in this hospital?
- As compared to your feeling about the expectations of the consortium? As compared to your own needs to understand this hospital?
- As compared to what you got from the other hospitals in the same country?
- What are your key remaining questions?
- Do you have any comments about the accuracy of the data you collected? Could there be any misinformation, why do you think so?

4.6 Did you your (or the team’s) ability to collect data change from one hospital to the next? How (for example asking questions differently, able to find right informants faster, etc)? What did you change?

**SECTION 5. FINAL REMARKS**

5.1 Do you have any comment or feedback on how people in this hospital are feeling about the ALERT project?

Probes:

- What were the questions or comments of the hospital people on the future intervention?
- Did respondents at the hospital ask you any questions about the project? What type?

5.2 From your impression, what is the likelihood of this hospital doing well with an intervention like ALERT? Why?

5.3 Is there anything else that you would like to mention about this hospital but I did not ask about?

*Thank you for your time and honest views. This is very useful to the whole ALERT project and making sure we support the participating hospitals as best as possible.*
